# Preparing Biochars from Cow Hair Waste Produced in a Tannery for Dye Wastewater Treatment

**DOI:** 10.3390/ma14071690

**Published:** 2021-03-30

**Authors:** Jinzhi Song, Yun Li, Yang Wang, Lei Zhong, Yang Liu, Xinyue Sun, Bo He, Yanchun Li, Shan Cao

**Affiliations:** 1 State Key Laboratory of Biobased Material and Green Papermaking, School of Light Industry and Engineering, Qilu University of Technology (Shandong Academy of Sciences), Jinan 250353, China; 1043118248@stu.qlu.edu.cn (J.S.); 1043118526@stu.qlu.edu.cn (L.Z.); 1043117266@stu.qlu.edu.cn (Y.L.); 1043118034@stu.qlu.edu.cn (X.S.); 1043119079@stu.qlu.edu.cn (B.H.); 2 College of Chemistry and Chemical Engineering, Yantai University, Yantai 264005, China; liyun@ytu.edu.cn; 3 College of Chemistry, Chemical Engineering and Materials Science, Shandong Normal University, Jinan 250014, China; wangyang@sdnu.edu.cn

**Keywords:** biochar, cow hair waste, keratin, high specific surface area, dye adsorption

## Abstract

A large amount of cow hair solid waste is produced in leather production, and a reasonable treatment should be developed to reduce the pollution. In this study, cow hair waste was utilized as the carbon precursor, and N_2_ was determined to be the most appropriate atmosphere for biochar preparation. We performed a comparison of the properties of biochars that were prepared with different methods, including direct pyrolysis, KOH activation, and the MgO template method. The characterization results show that the highest specific surface area reaches 1753.075 m^2^/g. Subsequently, the keratin that was extracted from cow hair and purified was used to prepare a biochar with the MgO template method, obtaining an orderly sponge structure. The biochar from cow hair waste was further used to absorb direct blue dye wastewater, and its adsorption capacity reached 1477 mg/g after 10 h with a high efficiency of regeneration. This study successfully utilized keratin-containing hair waste and provides a new source for synthesizing carbon materials for dye wastewater treatment.

## 1. Introduction

In leather production, a large amount of solid waste is produced. One of the main components of the waste is cow hair. Every ton of salt-wet cowhide can produce around 85 kg of waste hair [1]. Compared to wool, cow hairs are particularly difficult to apply in the textile industry due to their short length. The accumulation of large quantities of hair waste causes a huge waste of resources and deterioration of the environment, and it should be solved urgently. This hair waste is mainly composed of keratin, which is difficult to naturally degrade, and contains small amounts of lipids and minerals [2]. Only a few studies have paid attention to the recovery and reuse of cow hair waste, resulting in a huge waste of keratin resources. The keratin resources should be utilized for environmental benefit and economic value. Therefore, it is of great significance to find an effective method for the disposal of cow hair waste.

In recent years, bio-solid waste has been considered to be a carbon precursor for the preparation of biochars [3]. Successful attempts have been made to convert keratinous waste from the food industry and breeding industry, including feathers [4], ox horns [5], and pig nails [6]. Owing to the unique microscopic morphology, high specific surface area, and diverse elemental composition of biochars, increasing attention has been paid to potential applications in adsorption [7], electro-catalysis [8], and electrodes [9]. Ren et al. used goat hairs to achieve 3D porous carbon materials that were doped with nitrogen, oxygen, and phosphorus [10]. Based on these studies, natural keratin can be applied as a carbon precursor to prepare porous biochars with high performance. However, compared with the keratinous solid waste from the breeding industry and food industry, the cow hair waste from tanneries contains more impurities. It may influence the biochar preparation process.

Natural biomaterials have a unique structure and special composition, which will have an obvious impact on the final structure of prepared porous carbon materials [11]. The keratin formed by polypeptide chains constitutes the three-dimensional network structure of the cow hair [12]. By carbonization, the original hair structure will endow the obtained biochar with a special structure as well as high carbon content [13]. In addition, the biochar is usually heteroatom-doped, such as nitrogen-, oxygen-, or sulfur-doped [14]. In order to obtain a biochar with a high specific surface area and a well-distributed pore size, activators such as KOH [15], ZnCl_2_ [16], H_3_PO_4_ [17], and HNO_3_ [18] can be used to improve the properties. KOH activation can be considered because of its short activation cycle, simple operation, and excellent activation effect [19], and template methods may improve the orderliness of the mesoporous structure [20]. The biochar, which possesses abundant surface functional groups and a high specific surface area, may be favorable in application for adsorbing water contaminants [21].

In this study, the properties of cow hair waste produced in leather production are analyzed, and the original waste and keratin extracted from the waste are both used to prepare porous biochars. The influence of different carbonization atmospheres, including N_2_, Ar, and air atmospheres, on the properties of the biochar are explored for optimization, and KOH activation and the MgO template method are applied for further improving the properties. The control strategies that can affect the performance of biochar are analyzed and summarized. Then, the obtained biochar is used to treat direct blue dye wastewater. The adsorption ability and the regeneration effect are observed and evaluated. This study provides an effective method for turning hair waste into biochar and applying it for dye adsorption.

## 2. Materials and Methods

### 2.1. Materials

The cow hair waste and the direct blue dye (DB) were of industrial grade and collected from the hair-saving unhairing process in Baoen Leather Industry Co., Ltd. (Zibo, China). The WB600 keratinase was obtained by Cao’s method [22]. The granular activated carbon (AC) was of analytical grade and purchased from Sinopharm Group Chemical Reagent Co. Ltd. (Shanghai, China). Other reagents were of analytical grade and purchased from Tianjin Damao Chemical Reagent Factory (Tianjin, China).

### 2.2. Characterization of Cow Hair Waste

The cow hair waste was washed with distilled water (1/10, *v/v*) 3 times and dried at 80 °C until the weight was constant. The structure was observed by field emission scanning electron microscopy (FESEM; s4800, Hitachi, Tokyo, Japan), and the elements in the cow hair wastes were analyzed by Energy Disperse Spectroscopy (EDS).

### 2.3. Pre-Treatment of Cow Hairs

The cow hairs were washed with distilled water and dried in an air-circulating oven at 80 °C for 6 h. Then, the cow hairs were stored at 25 °C. The keratin was roughly extracted 10 times in NaOH solution (0.1 mol/L, *w/w*) at 100 °C for 2 h, and then the hair was deeply degraded by WB600 keratinase at the concentration of 160 U/mL at 60 °C for 6 h until completely dissolved. Then, the hydrolysate solution was dialyzed overnight and freeze-dried for 72 h.

### 2.4. Preparation of Cowhair-Based Char Materials

#### 2.4.1. The Carbonization

The cow hairs were carbonized directly. The cow-hair-based char materials (CCMs) were prepared in N_2_, Ar, and air atmospheres with a temperature-programmed heat treatment. The carbonization was processed at 350 °C for 1 h at a rate of 5 °C/min, and then the temperature was raised to 600 °C for 3 h. The samples obtained merely by the carbonization are tagged as CCMc. The obtained CCMcs under different hot coflow conditions are labeled as CCMc–N2, CCMc–Ar, and CCMc–Air, respectively.

#### 2.4.2. KOH Activation

After cooldown, 1.00 g of CCMc were grinded and sifted. Then, they were mixed with KOH at a ratio of 1:4.5 (*w/w*) in 30 mL of distilled water. The mixture was stirred at 45 °C for 18 h and dried at 110 °C. Subsequently, the samples were placed into the crucibles, and then were heated using the temperature-programmed heat treatment. The temperature rose continuously to 800 °C for 3.5 h at a rate of 5 °C/min. The cooled samples were washed with HCl (1.0 mol/L) to wipe off the residual KOH and then washed with deionized water until neutral pH. Finally, the samples were dried at 80 °C for 24 h. The finished CCMs are defined as CCMa–N_2_, CCMa–Ar, and CCMa–Air, respectively.

### 2.5. Preparation of Char Materials by the Template Method

The keratin powder was processed by the MgO template method [23]. The samples obtained using keratin were defined as keratin-based char material (KCM). A total of 30 mL of keratin solution (200 g/L) was mixed with 6 g of magnesium citrate in 200 mL of distilled water with stirring, and 50 mL of NaOH (2.5 mol/L) was dripped into the mixture. Then, the sample was stirred at 70 °C for 3 h and dried at 100 °C. The carbonization process was performed under a nitrogen atmosphere with a similar temperature-programmed heat treatment to that previously mentioned in the carbonization. Afterwards, the carbonized sample was pickled using HCl (10 wt%) to remove the MgO and washed with distilled water to pH 7. The sample was dried at 80 °C and is defined as KCMc–T. In addition, the keratin powder was carbonized directly as a control sample, and this product is defined as KCMc–C. Afterwards, the KCMc–T and KCMc–C were treated with KOH activation, and the samples are defined as KCMa–T and KCMa–C, respectively.

### 2.6. Characterization of Porous Carbon Materials

The morphology of CCMs and KCMs was observed with field emission scanning electron microscopy (FESEM; s4800, Hitachi, Tokyo, Japan) and transmission electron microscopy (TEM; JEM-2100, JEOL, Tokyo, Japan) [24]. An X-ray energy dispersive spectrometer (EDS) was used to perform the elemental analysis of samples under an SEM environment (Phenom pure plus, Phenom-World, Eindhoven, Netherlands). An X-ray diffractometer (XRD-6100, Shimadzu Corp., Kyoto, Japan) was used to analyze the crystalline structure of CCMs and KCMs. Raman spectroscopy (Horiban Jobin Yvon, LabRAM HR Evolution, Paris, France) was employed to investigate the Raman spectrum of the samples. FTIR spectra were collected on an IRAffinity-1S FTIR spectrometer (Shimadzu Corp., Kyoto, Japan) using the KBr pellet technique [15]. An ESCALABXi + X-ray photoelectron spectrometer (Thermo fisher technology co., LTD, Waltham, MA, USA) was used for elemental analysis. The specific surface area of the samples was evaluated by N_2_ absorption–desorption and the Brunauer-Emmett-Teller (BET) method using the Micromeritics Gemini 2380 (Micromeritics instrument Co., Norcross, GA, USA) surface area analyzer [25]. Before absorption measurements, the samples were degassed at 150 °C for 3 h in a vacuum. Then, the Barrett-Joyner-Halenda (BJH) theory method was used to determine the pore size distributions [26]. In addition, all the experiments were repeated at least three times.

### 2.7. Adsorption and Regeneration Experiments

#### 2.7.1. Adsorption Experiment

The conventional dosage of dye used in leather production is usually approximately 5% (*w/w*) of hide weight [27]. After dyeing, the dye concentration of wastewater is in the range of 10–50 g/L, which was referred to for designing the concentration of simulated direct blue dye wastewater. The AC is used for comparison. In addition, the saturated dye solution (100 g/L) was used for exploring the highest adsorption ability of biochar. A total of 50 mg of biochar was put into 10 mL dye solutions with different concentrations and shaken at 25 °C. The maximum absorption wavelength of the direct blue dye was determined by UV detection at 567 nm [28]. The adsorption capacity at each moment was calculated according to Equation (1).

(1) $$Q_{t}=\frac{C_{o}-C_{t}}{M}\cdot V$$

where *Q_t_* is the adsorption capacity at time *t* (mg/g); *C_o_* is the initial concentration of the direct blue dye (mg/L); *C_t_* is the ultimate concentration of the direct blue dye at time *t* (mg/L); *M* is the mass of the sample (g); and *V* is the volume of the dye solution (L).

#### 2.7.2. Desorption and Regeneration Experiment

After adsorption, the biochar was washed with 10 mL of a mixed solution of HNO_3_ (0.5 mol/L) and NaNO_3_ (0.5 mol/L) at 80 °C for 1 h. Then, it continued to be washed until the supernatant was transparent. The washed biochar was dried at 40 °C for 24 h and used for next round of adsorption. The adsorption experiment with a direct blue concentration of 50 mg/L was performed with 5 cycles.

## 3. Results

### 3.1. Properties Analysis of Cow Hair Waste

**Figure 1:**
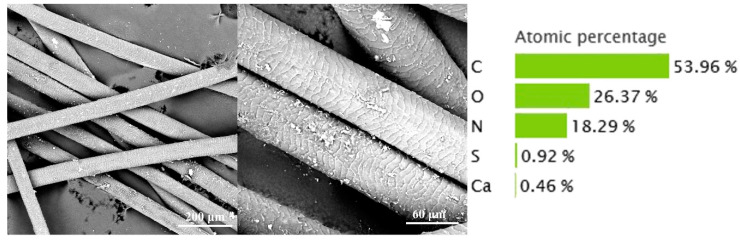
Image of hair waste.

The cow hair waste retained a relatively intact scale layer. After washing, Na_2_S and other reagents can be successfully removed. The C, N, and O elements are 53.96%, 18.29%, and 26.37% (Figure 1), respectively. The high C content is positive for preparing biochar. In addition, small amounts of S (0.92%) and Ca (0.46%) were detected in the cow hair, which are due to the presence of disulfide bonds in the keratin and the use of lime in the liming process, respectively.

### 3.2. The Preparation of Biochars

**Figure 2:**
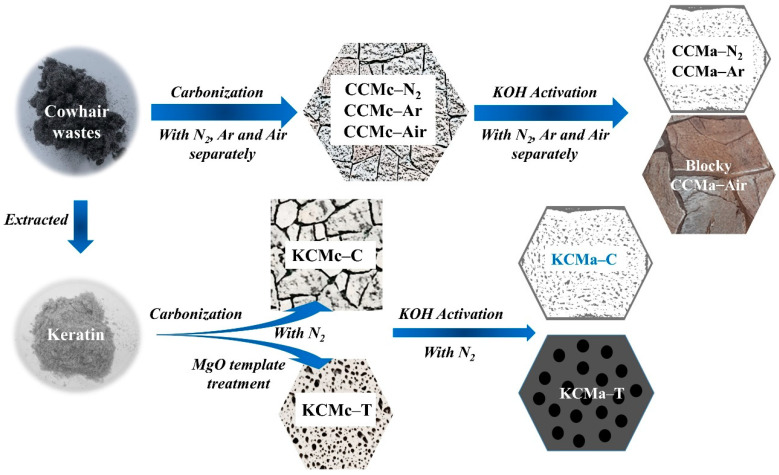
Flowchart of biochar preparation.

The flowchart of biochar preparation is described in Figure 2. Cow hair waste and keratin extracted from the waste were used for preparing biochars. The CCMcs (CCMc–N_2_, CCMc–Ar, and CCMc–Air) were obtained by carbonization at 350–600 °C with N_2_, Ar, and air, separately. After the KOH activation, the CCMa–N_2_ and CCMa–Ar with a similar bread-like structure were obtained, and CCMa–Air has blocky nanostructures. In addition, the keratin was extracted from cow hairs by NaOH and WB600 enzymes. The keratin-based biochars were prepared with an MgO pretreatment in an N_2_ atmosphere. The simple carbonization causes the compacted structure of KCMa–C, whereas the MgO template treatment endows the KCMa–T with an orderly porous sponge structure.

### 3.3. Element Composition Analysis of Biochars

**Figure 3:**
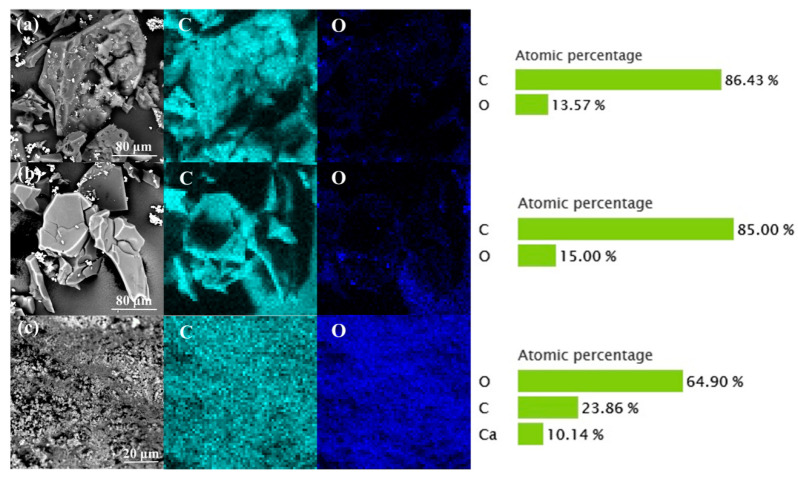
EDS of cow-hair-based char materials (CCMs). (**a**) CCMa–N_2_; (**b**) CCMa–Ar; and (**c**) CCMa–Air.

Cow-hair-based biochars carbonized in N_2_, Ar, and air were observed and analyzed and the results are shown in Figure 3. It shows that the elemental composition of CCMa–N_2_ and CCMa–Ar are similar and include C and O. The C content of CCMa–N_2_ and CCMa–Ar is 86.43% and 85%, respectively, along with significant O-doping. The CCMa–Air includes high content of O (64.9%) and Ca (10.14%). It is known that hair waste usually contains some lime due to the dehair-liming process in leather production. Some CaCO_3_ is produced with the presence of O_2_ in air in carbonization, and it is difficult to fully remove it by HCl washing in KOH activation [29].

### 3.4. Morphology Observation

#### 3.4.1. Morphology of CCMs

**Figure 4:**
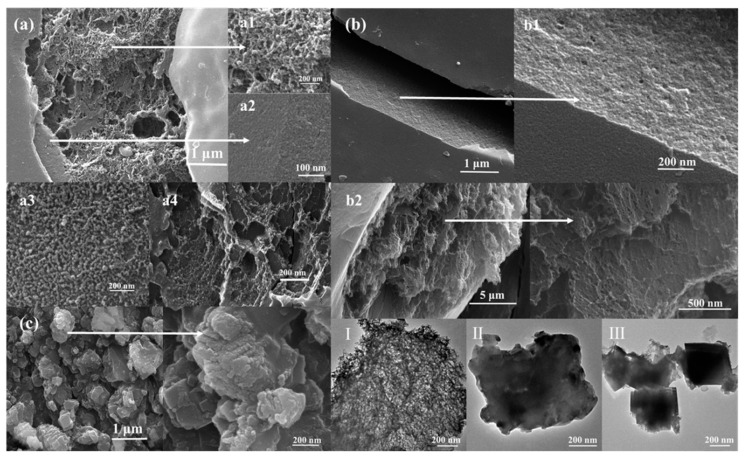
SEM (**a**–**c**) and TEM (I–III) images of (**a**) CCMa–N_2_; (**b**) CCMa–Ar; (**c**) CCMa–Air; (**I**) CCMa–N_2_; (**II**) CCMa–Ar, and (**III**) CCMa–Air.

The morphology of activated porous biochars was further observed by SEM. As shown in Figure 4, CCMa–N_2_ had a bread-like structure. The external structure of CCMa–N_2_ contained a smooth and flat outer shell (Figure 4(a2)), whereas its interior possessed a sponge structure (Figure 4(a1)). The internal pore diameters were irregular in the range of 5 nm (Figure 4(a3)) to 50 nm (Figure 4(a4)). In carbonization, the original hair structure is cracked. The tightly overlapping scales on the cow hair surface disappear at a high temperature to form the shell with a smooth surface. The organic components in the cow hair fibers are cracked, and C=O and hydrogen-containing functional groups are volatilized to produce the larger pore structure in the carbon materials. The KOH activation produces the internal sponge structure, and the oxidation reaction of KOH produces H_2_, CO_2_, and CO gases, which cause the pore structure [30].

With the Ar atmosphere, the interior structure of the obtained biochar (Figure 4(b2)) is similar to the structure of CCMa–N_2_. A large number of pores appear to form the sponge structure, which should be attributed to KOH activation, and it effectively improves the specific surface area of CCMa–N_2_ and CCMa–Ar [31]. As shown in Figure 4c, irregularly shaped particles were produced with the air atmosphere. With high-temperature pyrolysis, the reaction between O_2_ in air and keratin results in the loss of C-containing functional groups of produced biochar, thereby contributing to the morphology with small and inconsistent size particles. By TEM spectra analysis (Figure 4I–III), the bread-like structures of CCMa–N_2_ and CCMa–Ar can be scrutinized with the abundance of interior pores, and the distribution of pores in CCMa–Ar is tighter than that in CCMa–N_2_.

#### 3.4.2. Morphology of KCMs

**Figure 5:**
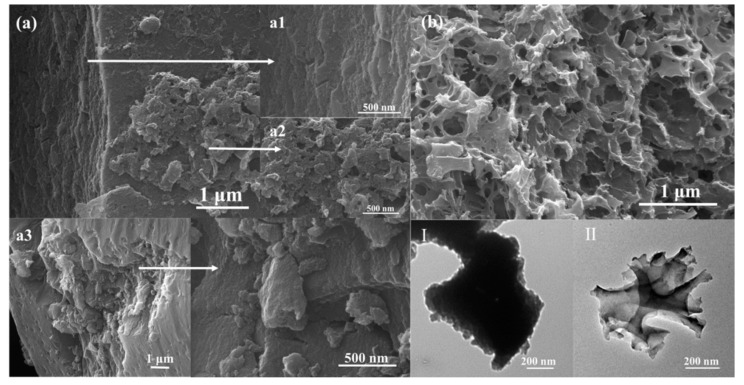
SEM (**a**–**b**) and TEM (**I–II**) images of (**a**) keratin-based char material (KCM)a–C; (**b**) KCMa–T; (**I**) KCMa–C; and (**II**) KCMa–T.

The KCMa–C is blocky with a large scale (Figure 5(a1)), and the KOH activation produces a number of pores in the surface (Figure 5(a2)). By the hydrolysis of hair to extract keratin, polypeptide chains with different molecular weights are generated, forming different scales of block structures (Figure 5(a3)). Obviously, the MgO template method leads to an orderly sponge structure (Figure 5b). After pyrolysis, MgO is removed, and the originally occupied position forms the mesoporous structure. In the TEM spectra (Figure 5I,II), the structure of KCMa–C is more compacted (Figure 5I). A number of mesopores appear by the MgO template method in KCMa–T (Figure 5II).

### 3.5. BET Analysis

**Figure 6:**
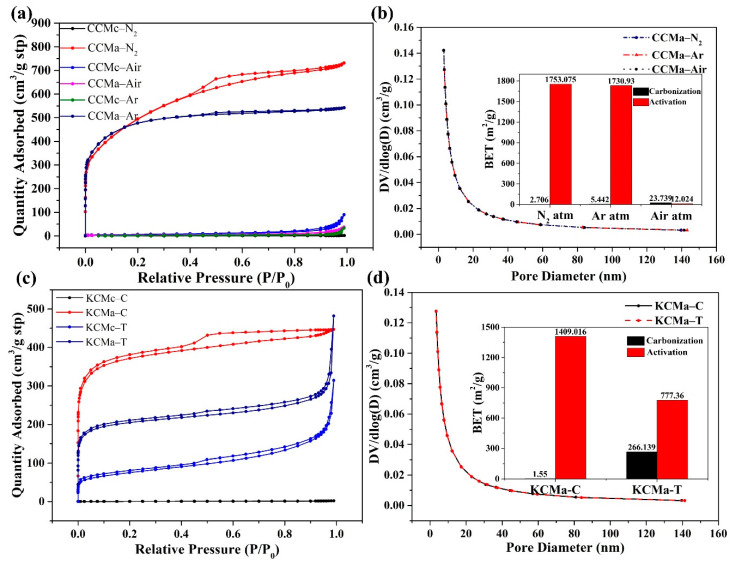
(**a**) N_2_ adsorption-desorption isotherm of CCMs; (**b**) Pore size distribution curves of CCMs; (**c**) N_2_ adsorption-desorption isotherm of KCMs and (**d**) Pore size distribution curves and Brunauer-Emmett-Teller (BET) values of KCMs.

The N_2_ adsorption–desorption isotherms and the corresponding pore size distribution curves of biochars, which were prepared under different atmospheres, were used to analyze the porous structure and specific surface area. In Figure 6, similar type IV absorption and desorption isotherms along with an H4 hysteresis curve [32] appear for CCMa–N_2_, CCMa–Ar, KCMa–C, KCMc–T, and KCMa–T. This indicates that the accumulated particles form slit pores, which produce mesoporous structures in biochar [33]. The specific surface area of CCMa–N_2_ and CCMa–Ar reaches 1753.075 and 1730.93 m^2^/g (Table S1), respectively, representing that the KOH activation increases the specific surface area effectively. The specific surface area of KCMa–C is 1409.016 m^2^/g. Though the MgO template method can make the biochar structure more orderly, the specific surface area is not improved. This is because MgO forms a large number of macroporous structures, and the increasing pore volume leads to a decrease in the specific surface area of KCMc–T (Figure 5). By observing the pore diameter distribution, the diameters of CCMs and KCMs were found to mainly be in the range of 2–20 nm, which presents the appearance of a large number of mesopores [34]. The unique porous structure and high specific surface area of biochar may result in an abundance of applications, such as adsorption materials [35] and electrode materials [36], in the future.

### 3.6. Characterization

#### 3.6.1. XPS Analysis

**Figure 7:**
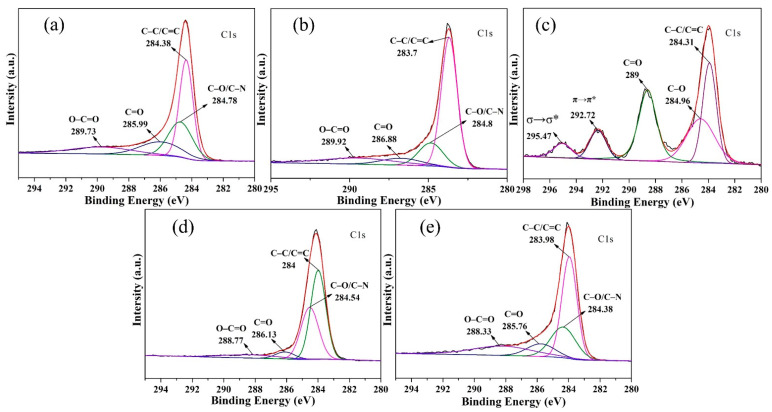
XPS spectra (C1s) of (**a**) CCMa–N_2_; (**b**) CCMa–Ar; (**c**) CCMa–Air; (**d**) KCMa–C and (**e**) KCMa–T.

Figure 7a–c displays the XPS spectra of CCMa–N_2_, CCMa–Ar, and CCMa–Air. In the C1 s spectra of CCMa–N_2_ (Figure 7a), the dominant peaks at 284.38, 284.78, 285.99, and 289.75 are attributed to C–C/C=C, C–O/C–N, C=O, and O–C=O, respectively [37]. The C–C/C=C peak intensity of CCMa–Ar (Figure 7b) decreases to 283.7 eV, whereas the C–O/C–N peak intensity is at 284.8 eV. This suggests the dehydrogenation of keratin and the formation of O-doped CCMa–N_2_ under the N_2_ atmosphere. From the C1 s peaks of CCMa–Air (Figure 7c), the signals at 292.72 eV and 295.47 eV should be assigned to π and σ electron delocalization, respectively [38]. The increased C=O peak intensity and the disappearance of O–C=O are due to the reaction between the cow hair and the oxygen in the air. From the C1 s spectra of KCMa–C (Figure 7d) and KCMa–T (Figure 7e), the peaks around at 284, 284.4, 286, and 288.5 eV represent C–C/C=C, C–O/C–N, C=O, and O–C=O, respectively. Therefore, the amounts of amides and hydrogen-containing functional groups decrease, and a number of oxygen-containing functional groups remain in the biochar in the N_2_ and Ar atmospheres (Figure S2). The oxygen content of biochar is significant in application, and it has been confirmed that oxygen-containing functional groups have important effects on the absorption of mercury [39] and the capacitive properties of biochars [40].

#### 3.6.2. XRD and Raman analysis

**Figure 8:**
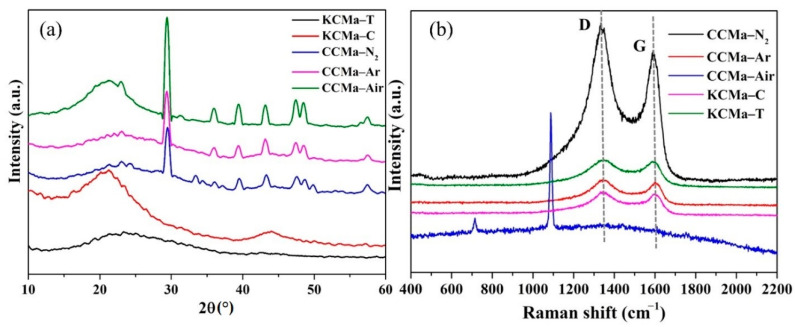
(**a**) XRD spectra and (**b**) Raman spectra.

The XRD spectra of CCMs and KCMs are displayed in Figure 8a, which shows an amorphous structure. CCMa–Air has a wide diffraction peak at 21°, reflecting the presence of amorphous or disordered carbon [41]. In the spectra of CCMs, the diffraction peaks appear at 23 and 43°, corresponding to the (002) (parallel graphite flake accumulation) and (100) lattice planes of graphite, respectively [42]. This indicates the formation of sp2 hybrid carbon, which reflects that these chars contain single-layer carbon sheet structures. The wide peaks at 23 and 43° in CCMa–N_2_, CCMa–Ar, and CCMa–Air represent the existence of amorphous carbon. The peaks of CCM at 29.4 and 36° indicate the 110 and 111 lattice planes of carbon, respectively [43]. In addition, the peaks of CCM at 39.5, 47.5, 48.5, and 57.5° represent the crystal phases of the different keratin-based biochars. The diffraction peaks at 39.5, 47.5, 48.5, and 57.5 correspond to the Bragg reflection planes of 107, 206, 304, and 314, respectively [44]. Both KCMa–C and KCMa–T have a wide diffraction peak in the range of 20–30°. This indicates that KCMa–C and KCMa–T are disordered graphite carbon. The wide diffraction peak of KCMa–C at 44° further proves the amorphous structure [45].

Figure 8b shows the Raman spectra of CCMs and KCMs. The peaks of the G band and D band of these biochars are exhibited at 1343 cm^−1^ and 1603 cm^−1^. The D-band represents the low symmetry or irregularity of carbon materials, while the G-band is generated by the stretching vibration of sp2 hybrid atoms in the sample, indicating the presence of ordered graphitized carbon [46]. The intensity ratio of the D-band to the G-band (ID/IG) indicates the graphitization degree of carbon [47]. The ID/IG of CCMa–N_2_ and CCMa–Ar is 1.11 and 1.028, respectively. This indicates that the graphitization degree of CCMa–N_2_ is lower than that of CCMa–Ar. The CCMa–Air does not have a graphitized structure [48] because the presence of O_2_ in air leads to a decrease in the C content in the final biochar. The new peaks around 700 and 1100 cm^−1^ of CCMa–Air are attributed to the residual calcium [49]. In addition, the ID/IG of KCMa–C and KCMa–T is 1.027 and 1.017, respectively. This indicates that the graphitization degree of KCMa–T is successfully improved by the MgO template method.

### 3.7. Adsorption and Regeneration Experiments

**Figure 9:**
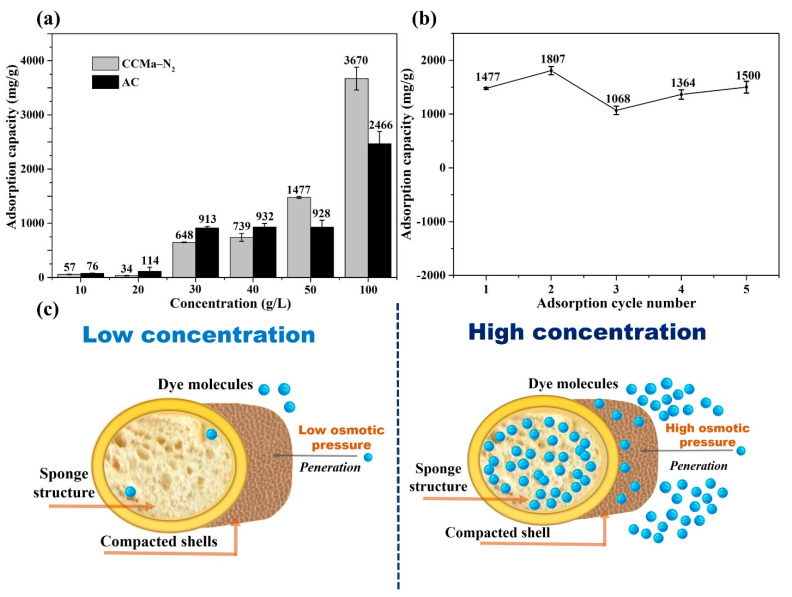
Adsorption and regeneration. (**a**) The adsorption capacity analysis; (**b**) The regeneration capacity in 50 g/L of direct blue (DB) dye solution; (**c**) The adsorption mechanism diagram.

The results show that the adsorption capacity of CCMa–N_2_ to DB dye reaches 1477 mg/g in 50 g/L of DB solution, while the adsorption capacity of AC is 928 mg/g. Thereby, the CCMa-N_2_ shows a higher adsorption capacity with a high concentration of wastewater. When the DB concentration has a low concentration (below 40 g/L), the adsorption capacity is low, which should result in the special bread-like structure. The compacted shell of biochar prevents the penetration of dye molecules as shown in Figure 9c. Compared to CCMa–N_2_, the structure of AC is open-type, which can take the physical adsorption with the dye in wastewater sufficiently at low concentrations [50]. The adsorption capacity of AC can reach 932 mg/g in 40 g/L of DB solution, while that of CCMa–N_2_ is 739 mg/g. With the increase in the dye concentration in solution, the osmotic pressure is improved. The high driving force causes a large amount of dye molecules to penetrate through the compacted shell [51], while the compacted shell prevents the spread of dye molecules in solution. Thus, the dye molecules can be adsorbed onto the surface of the compacted shell as well as in the spongy interior (Figure 9c). In the saturated solution of the DB dye, the adsorption capacity reaches 3670 mg/g (Figure 9a), which is higher than that of AC (2466 mg/g). In addition, the desorption of dye and the regeneration adsorption of biochar are significant as shown in Figure 9b. In five cycles, the absorption ability of CCMa–N_2_ is stable at around 1500 mg/g. In summary, the keratin-based biochars display a positive ability to adsorb dye, and they can be expected to be used for further heavy metal adsorption with a high concentration.

## 4. Conclusions

In this study, cow hair waste was used to prepare porous biochar due to the integral structure and high carbon content. The formation mechanism of biochar based on hair waste was analyzed under different atmospheres. The porous biochars including CCMa–N_2_ and CCMa–Ar have a similar and complex bread-like structure and a high degree of graphitization. The specific surface area of CCMa–N_2_ reaches 1753.075 m^2^/g. The KCMa–T, which was generated by the MgO template method, has an orderly sponge structure with a large number of mesopores, and has the highest graphitization degree (ID/IG = 1.017). The purified keratin extracted from the hair waste leads to the simple structure of KCMa–T and KCMa–C, and the specific surface area of KCMa–C is 1409.016 m^2^/g. The CCMa-N_2_ is effective for DB dye adsorption and its adsorption ability in 50 g/L of DB dye solution (1477 mg/g) is better than that of activated carbon (928 mg/g). In five cycles of regeneration, the adsorption ability was found to be stable at approximately 1500 mg/g. This study proposes a novel method to deal with the cow hair solid waste produced by the leather industry and provides a highly efficient adsorbent for further applications.

## Data Availability

Data is contained within the article.

## Supplementary Materials

The following are available online at https://www.mdpi.com/article/10.3390/ma14071690/s1, Table S1: The BET of biochars. Figure S1: XPS spectra of (**a**) CCMa–N_2_, (**b**) CCMa–Ar, (**c**) CCMa–Air, (**d**) KCMa–C, and (**e**) KCMa–T. Figure S2: FTIR spectra of (**a**) CCMs and (**b**) KCMs.

## Abbreviations

**Full Name**	**Abbreviation**
Cowhair-based char material-carbonization	CCMc
Cowhair-based char material-activation	CCMa
Keratin-based char material-carbonation	KCMc
Keratin-based char material-activation	KCMa
