# Supplementary material for: Preparing Biochars from Cow Hair Waste Produced in a Tannery for Dye Wastewater Treatment
Source: Materials (Basel). 2021 Mar 30;14(7):1690. doi: 10.3390/ma14071690 (PMC8036782; doi:10.3390/ma14071690)
Supplement: Supplementary file 1 [file materials-14-01690-s001.pdf]

materials

MDPI

*Supplementary Material*

# Preparing Biochars from Cow Hair Waste Produced in a Tannery for Dye Wastewater Treatment

**Jinzhi Song [1], Yun Li [2], Yang Wang [3], Lei Zhong [1], Yang Liu [1], Xinyue Sun [1], Bo He [1], Yanchun Li [1,*], and Shan Cao [1,*]**

1 State Key Laboratory of Biobased Material and Green Papermaking, School of Light Industry and Engineering, Qilu University of Technology (Shandong Academy of Sciences), Jinan 250353, China; 1043118248@stu.qlu.edu.cn (J.S.); 1043118526@stu.qlu.edu.cn (L.Z.); 1043117266@stu.qlu.edu.cn (Y.L.); 1043118034@stu.qlu.edu.cn (X.S.); 1043119079@stu.qlu.edu.cn (B.H.)
2 College of Chemistry and Chemical Engineering, Yantai University, Yantai 264005, China; liyun@ytu.edu.cn
3 College of Chemistry, Chemical Engineering and Materials Science, Shandong Normal University, Jinan 250014, China; wangyang@sdnu.edu.cn
* Correspondence: lyc@qlu.edu.cn (Y.L.); cs1988@qlu.edu.cn (S.C.)

**Citation:** Song, J.; Li, Y.; Wang, Y.; Zhong, L.; Liu, Y.; Sun, X.; He, B.; Li, Y.; Cao, S. Preparing Biochars from Cow Hair Waste Produced in a Tannery for Dye Wastewater Treatment. *Materials* **2021**, *14*, 1690. https://doi.org/10.3390/ma14071690

Academic Editors: Dorota Kołodyńska, Teofil Jesionowski and Polycarpos Falaras

Received: 5 January 2021
Accepted: 20 March 2021
Published: 30 March 2021

**Publisher's Note:** MDPI stays neutral with regard to jurisdictional claims in published maps and institutional affiliations.

**Copyright:** © 2021 by the authors. Licensee MDPI, Basel, Switzerland. This article is an open access article distributed under the terms and conditions of the Creative Commons Attribution (CC BY) license (http://creativecommons.org/licenses/by/4.0/).

## 1. BET Analysis

The BET analysis of all samples is shown in Table S1, and it is seen that KOH activation can obviously improve the specific surface area of samples.

**Table S1.** The BET of biochars.

| Biochar | **BET ($m^2/g$)** |
|---|---|
| CCMc–$N_2$ | 2.706 |
| CCMa–$N_2$ | 1753.075 |
| CCMc–Ar | 5.442 |
| CCMa–Ar | 1730.93 |
| CCMc–Air | 23.739 |
| CCMa–Air | 12.024 |
| KCMc–C | 1.55 |
| KCMa–C | 1409.016 |
| KCMc–T | 266.139 |
| KCMa–T | 777.36 |

## 2. XPS Analysis

Survey spectra and O1s,N1s spectra were presented in Figure S1. In the survey spectra, there are four distinct elements in CCMa-$N_2$, which are C1s, O1s, N1s, S2p with the content of 87.96, 7.73, 2.23, 1.06%, respectively. And the CCMa–Ar has the similar proportion of elements. Because of the reaction between air and cow hair, the elements have significant changes, especially nitrogen and oxygen.

In the XPS spectra of O1s, there are three components including C=O, C–O/C–O–C and O–C=O in the CCMa–$N_2$ and CCMa–Ar [1]. However, the O-C=O in the CCMa-Air disappeared. This finding can be explained by the reaction between O–C=O with oxygen, resulting the O–C=O transformed into C=O. The CCMa–Air has the highest oxygen content, and the content of other samples are mainly carbon. The three component peaks are identified as pyridinic–N, pyrollic–N and graphitic–N at 397.7, 399.7 and 402 eV in CCMa–$N_2$ [2]. The CCMa–Ar has the same N1s peaks. Affected by the pyrolysis atmosphere, the CCMa–$N_2$ only has the pyrollic–N at 399 eV.

The results in survey spectra of Figure S1d-e showed that both KCMa-C and KCMa-T are extremely high in the content of C element. The peaks of C=O, C–O/C–O–C and O–C=O appear in the KCMa-C and KCMa-T. In the N1s spectra, the two samples only has pyrollic-N.

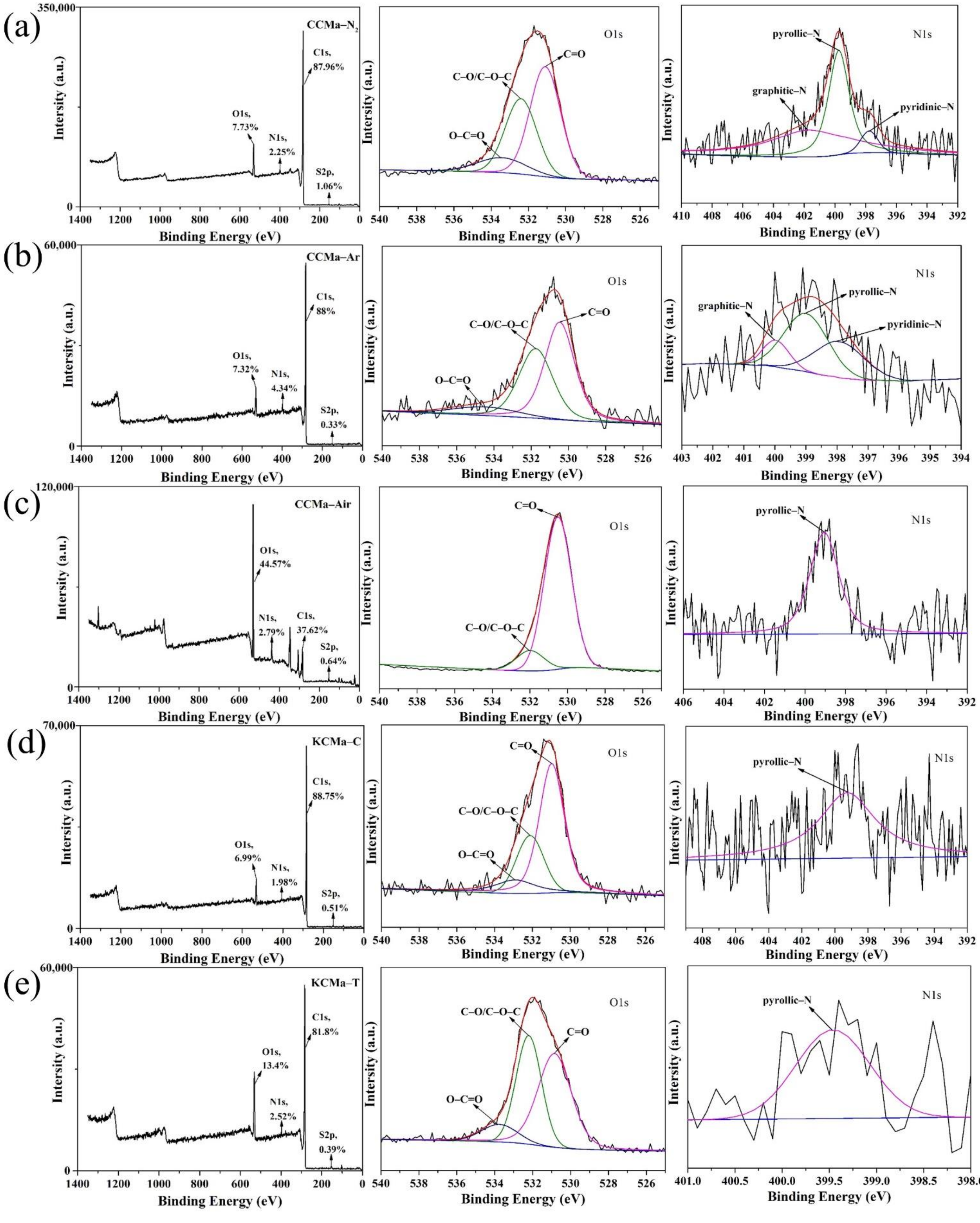


**Figure S1.** XPS spectra. (**a**) CCMa–N2; (**b**) CCMa–Ar; (**c**) CCMa–Air; (**d**) KCMa–C and (**e**) KCMa–T.

## 3. FTIR Analysis

The changes of chemical bonds in functional groups of CCM and KCM are analyzed by FTIR (Figure S2). The decrease of a broad peak intensity at 3400–3500 $cm^{-1}$ indicates

the break of N–H bonds in amine groups and O–H bonds in hydroxyl groups by pyrolysis [3]. The C–H stretching band is at 3075 $cm^{-1}$, and the peaks at 2915 and 2945 $cm^{-1}$ are attributed to $CH_2$ asymmetric and symmetric stretching bands, respectively [4]. The peaks of C–H and $CH_2$ disappear, and peak intensities of C=C [5] and C≡C [6] significantly increase, representing dehydrogenation of keratin. In addition, the peaks at 668, 870, 1648 $cm^{-1}$ represent the oxygen-containing functional group [7]. Due to the breakage of a number of C=O bonds, the intensities of C=O peak of CCMs and KCMs decrease obviously at 1648 $cm^{-1}$. A small amount of –OH groups presented on 870 $cm^{-1}$ are remained in CCMs, but not appear in KCMs. It is attributed to the breakage of hair structure during the extraction of keratin. The decrease of C–N stretching vibration peak at 1426 $cm^{-1}$ and N–H peak intensity at around 1516 $cm^{-1}$ indicates that the denitrification is performed in carbonization [8]. Above all, at high temperatures, large amounts of amides and hydrogen-containing functional groups decrease or disappear. The dehydrogenation and denitrification of keratin promote the formation of amorphous carbon structure of biochars.

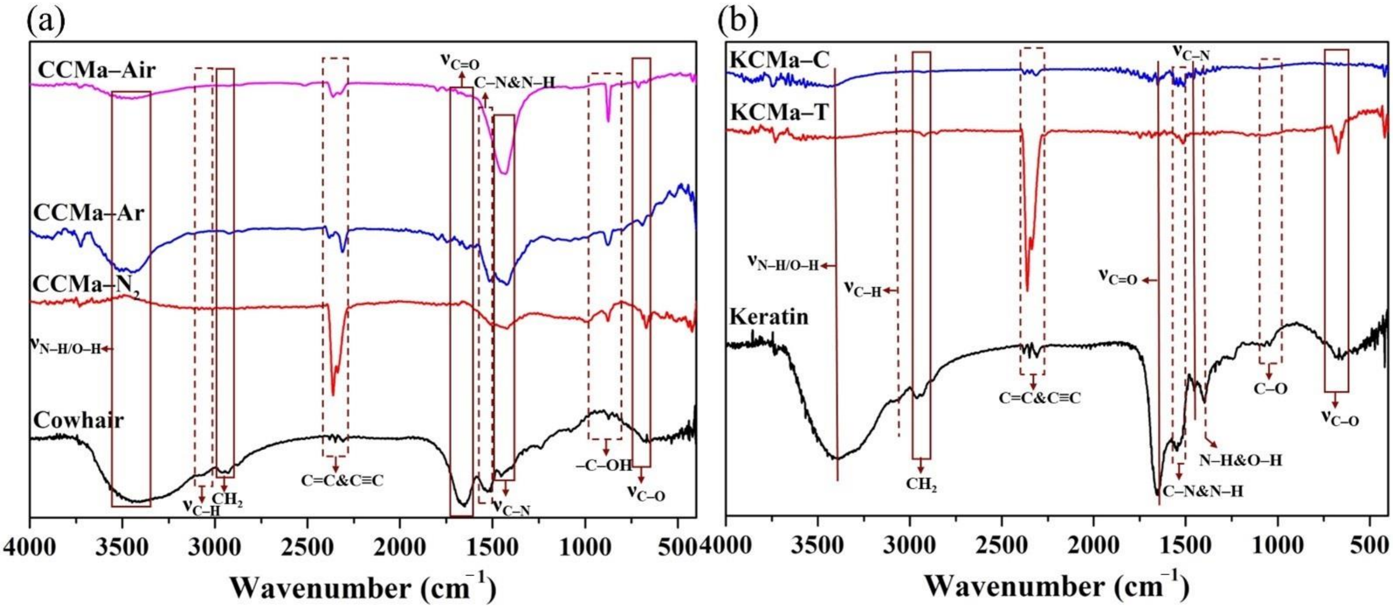


**Figure S2.** FTIR spectra of (**a**) CCMs and (**b**) KCMs.

## References

1. Saravanan, K.; Kalaiselvi, N. Nitrogen containing bio-carbon as a potential anode for lithium batteries. *Carbon* **2015**, *81*, 43–53, doi:10.1016/j.carbon.2014.09.021.
2. Zhu, J.; Li, W.; Li, S.; Zhang, J.; Zhou, H.; Zhang, C.; Zhang, J.; Mu, S. Defective N/S-Codoped 3D Cheese-Like Porous Carbon Nanomaterial toward Efficient Oxygen Reduction and Zn–Air Batteries. *Small* **2018**, *14*, 1800563, doi:10.1002/smll.201800563.
3. Alabadi, A.; Razzaque, S.; Yang, Y.; Chen, S.; Tan, B. Highly porous activated carbon materials from carbonized biomass with high $CO_2$ capturing capacity. *Chem. Eng. J.* **2015**, *281*, 606–612, doi:10.1016/j.cej.2015.06.032.
4. Mer, K.; Sajjadi, B.; Egiebor, N.O.; Chen, W.Y.; Mattern, D.L.; Tao, W. Enhanced degradation of organic contaminants using catalytic activity of carbonaceous structures: A strategy for the reuse of exhausted sorbents. *J. Environ. Sci.* **2021**, *99*, 267–273, doi:10.1016/j.jes.2020.06.030.
5. Huang, Z.; Shao, H.; Huang, B.; Li, C.; Huang, Y.; Chen, X. High-performance fish-scale-based porous carbon for the removal of methylene blue from aqueous solution. *RSC Adv.* **2014**, *4*, 18737–18743, doi:10.1039/C4RA00756E.
6. Konikkara, N.; Kennedy, L.J.; Vijaya, J.J. Preparation and characterization of hierarchical porous carbons derived from solid leather waste for supercapacitor applications. *J. Hazard. Mater.* **2016**, *318*, 173–185, doi:10.1016/j.jhazmat.2016.06.037.
7. Liu, H.; Ning, W.; Cheng, P.; Zhang, J.; Wang, Y.; Zhang, C. Evaluation of animal hairs-based activated carbon for sorption of norfloxacin and acetaminophen by comparing with cattail fiber-based activated carbon. *J. Anal. Appl. Pyrolysis* **2013**, *101*, 156–165, doi:10.1016/j.jaap.2013.01.016.
8. Cretescu, I.; Lupascu, T.; Buciscanu, I.; Balau-Mindru, T.; Soreanu, G. Low-cost sorbents for the removal of acid dyes from aqueous solutions. *Process. Saf. Environ. Prot.* **2017**, *108*, 57–66, doi:10.1016/j.psep.2016.05.016.
